# VolumePeeler: a novel FIJI plugin for geometric tissue peeling to improve visualization and quantification of 3D image stacks

**DOI:** 10.1186/s12859-023-05403-z

**Published:** 2023-07-12

**Authors:** Marilyn Gatica, Carlos F. Navarro, Alejandro Lavado, German Reig, Eduardo Pulgar, Paula Llanos, Steffen Härtel, Andrea Ravasio, Cristina Bertocchi, Miguel L. Concha, Mauricio Cerda

**Affiliations:** 1 NIHR Nottingham Biomedical Research Centre, School of Medicine, University of Nottingham, Nottingham, UK; 2 Integrative Biology Program, Institute of Biomedical Sciences, Facultad de Medicina, Universidad de Chile, Santiago, Chile; 3 Biomedical Neuroscience Institute, Santiago, Chile; 4 Escuela de Tecnología Médica and Centro Integrativo de Biología y Química Aplicada, Universidad Bernardo O’Higgins, Santiago, Chile; 5 CEDAI Aquaculture, Santiago, Chile; 6 Facultad de Ingeniería y Ciencias, Universidad Adolfo Ibáñez, Viña del Mar, Chile; 7 Center for Medical Informatics and Telemedicine, Facultad de Medicina, Universidad de Chile, Santiago, Chile; 8 Institute for Biological and Medical Engineering, Schools of Engineering, Medicine and Biological Sciences, Pontificia Universidad Católica de Chile, Santiago, Chile; 9 Laboratory for Molecular Mechanics of Cell Adhesion, Department of Physiology, Faculty of Biological Sciences, Pontificia Universidad Católica de Chile, Santiago, Chile; 10 Graduate School of Engineering Science, Osaka University, Osaka, Japan; 11 Center for Geroscience, Brain Health and Metabolism, Santiago, Chile

**Keywords:** Microscopy, Image processing, 3D projections, Virtual 3D peeling

## Abstract

**Motivation:**

Quantitative descriptions of multi-cellular structures from optical microscopy imaging are prime to understand the variety of three-dimensional (3D) shapes in living organisms. Experimental models of vertebrates, invertebrates and plants, such as zebrafish, killifish, *Drosophila* or *Marchantia*, mainly comprise multilayer tissues, and even if microscopes can reach the needed depth, their geometry hinders the selection and subsequent analysis of the optical volumes of interest. Computational tools to “peel” tissues by removing specific layers and reducing 3D volume into planar images, can critically improve visualization and analysis.

**Results:**

We developed VolumePeeler, a versatile FIJI plugin for virtual 3D “peeling” of image stacks. The plugin implements spherical and spline surface projections. We applied VolumePeeler to perform peeling in 3D images of spherical embryos, as well as non-spherical tissue layers. The produced images improve the 3D volume visualization and enable analysis and quantification of geometrically challenging microscopy datasets.

**Availability:**

ImageJ/FIJI software, source code, examples, and tutorials are openly available in https://cimt.uchile.cl/mcerda

**Supplementary Information:**

The online version contains supplementary material available at 10.1186/s12859-023-05403-z.

## Introduction

Recent advances and lower costs in optical microscopy have made 3D imaging accessible in many biological research laboratories worldwide. Yet, how to visualize and quantify these 3D volumes remains a challenge, especially when complex geometries are involved. To simplify visualization and quantification, cartographic projections [1], and depth-of-interest detection in large image stacks [2] have been proposed. However, cartographic views are often unfamiliar to biologists, which severely restricts their applicability. Methods for automatic detection of the depth of interest in image stacks (for instance, the LocalZProjector FIJI plugin [2]) implement specific criteria to select volume sections (such as local image sections), but in doing so they exclude other types of planes of interest, inherent to diverse research questions. More general algorithms, such as the ImSAnE MATLAB tool [3], allow to define an arbitrary volume of interest. However, such approaches require a segmentation of the surface of interest as input, which is not always available or easy to provide.

Experimental models for microscopy imaging, such as zebrafish and annual killifish embryos, present significant advantages in optical clarity and short life cycles. However, their sphere-like geometry is challenging for visualization and separation of tissue sections at different depths. Developing embryos already comprise several layers of non-flat tissue, and imaging can generate large data volumes. A plant model is *Marchantia*, which exhibits a rapid life cycle, and vegetative structures called gemmae, with a simple, easy-to-image, architecture. Gemmae have cells actively dividing in the notch area, allowing the acquisition of numerous high resolution time-lapse sequences of dividing cells. Yet, *Marchantia* cells layers have non-flat geometries, and visual inspection and assessment of cells and volumes of interest become difficult.

**Fig. 1:**
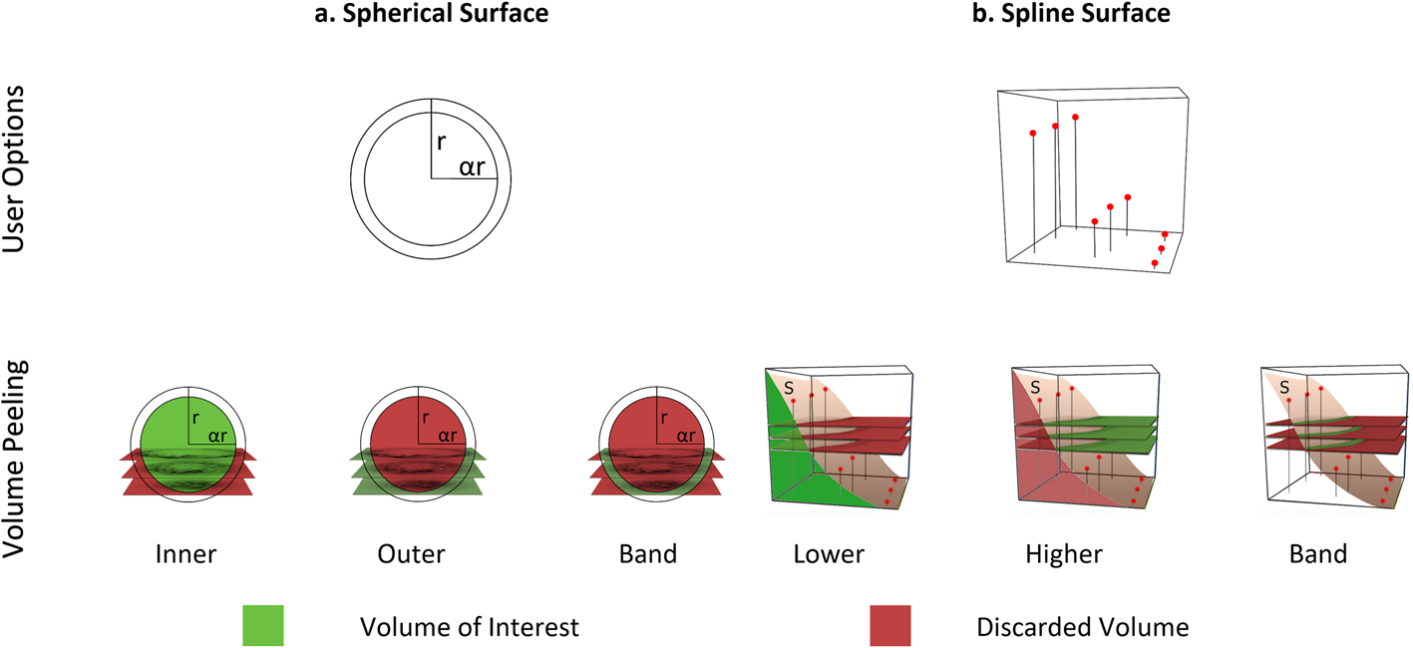
For the two available Spherical (**a**) and Spline (**b**) surface projections in VolumePeler, user parameter options (first row), and volume peeling options (second row) are depicted

In fluorescence microscopy, z-planes capture images at different focal planes along the z-axis. These z-planes enable both acquisition and visualization of three-dimensional (3D) structures within a sample. By acquiring images at multiple z-planes, researchers can reconstruct a comprehensive 3D representation of the sample, which enables detailed analysis and visualization of various structures and features. In this context, annual killifish, zebrafish, and *Marchantia* have a characteristic depth per location. A simple 2D projection of the three-dimensional volume can be made, taking the maximum intensity values along all z-planes, but only the brightest voxels will be visible. A selection of z-planes could be used, but as the layers’ depth depends on relative location within the sample, manual implementation becomes cumbersome. Geometrically, in all the above mentioned cases the problem arises because of the tissue curvature. To tackle this issue, we propose a geometric approach to perform volume cleaning and peeling in 3D image stacks, aimed to improve visualization and to allow usage of available 2D tools, as shown in Figure 1a.

To explain the application of our method, we focus on two main geometries: spherical surfaces, and user-guided spline surfaces (Figure 1). The effectiveness of our method is demonstrated first in a synthetic volume, and then by comparing the segmentation obtained after volume peeling with the corresponding manual segmentation. The proposed approach is available as a FIJI plugin [4, 5].

## Methods

We assume that the tissue to project can be either a spherical surface or a surface that can be represented by cubic spline functions. Once the surface is estimated, the volume is cleaned and projected into 2D. An overview of the method is shown in Figure 1, and a block diagram is provided in Additional file 1: Fig. 1.

### Spherical surface

To define the spherical surface projection, the inputs are an image volume *I* and a ratio parameter $\alpha$, that are used to fit a sphere within *I*. We define the optimization function as in [6],

1 $$\begin{aligned} \min _{(x_0,y_0,z_0,r_0)}\displaystyle \sum _{i \in N} \left| (X_i-x_0)^2+(Y_i-y_0)^2+(Z_i-z_0)^2-r_0^2 \right| , \end{aligned}$$

where $N =\{i; \ I(V_i) > \tau \}$, $V_i$ is the voxel at the position $(X_i,Y_i,Z_i)$ in the image *I*, $I(V_i)$ its intensity, and $\tau$ is an automatic threshold value, calculated using the Otsu’s method [7]. Solving the minimization problem (1) yields a sphere with radius $r_0$ centered at $(x_0,y_0,z_0)$. The parameter $\alpha$ is a value between 0 and 1 representing a percentage of the radius $r_0$ (Figure 1a).

To compute the peeling, we define a 3D binary image *T* such that each voxel value is 1 if $(X_i-x_0)^2+(Y_i-y_0)^2+(Z_i-z_0)^2\le (\alpha r_0)^2$, and 0 otherwise (Figure 1a, inner volume case). The peeled volume is given by $I^p=I\cdot T$.

### Spline surface

For the spline surface use case, we require as input the image *I* and user-defined control points to fit surfaces that a cubic spline can represent. The choice to manually set the control points allows the experts to define the depth of interest instead of a specific image feature (Figure 1b). The user is required to observe the specific (*x*, *y*)-coordinates in *I* and manually enter the corresponding *z*-value to visualize the layer of interest. The (*x*, *y*)-coordinates are defined as $(x_k,y_n)$ in a regular grid that allows 9, 16, or 25 points. For each $(x_k,y_n)$-coordinate, the parameter $z_i$ is a value between 1 and the image depth. With the manual control points $(x_k,y_n)$, a 2D cubic spline interpolation is computed to produce a function *S* that describes the surface.

To compute the peeling, we defined a binary image *T* such that each voxel is 1 if $S(X_i,Y_i)\le Z_i$ and 0 otherwise (Figure 1b, lower volume case). As before, the peeled volume is given by $I^p=I\cdot T$.

### 2D projection and additional features

Once the volume is peeled (either with the spherical or the spline surface), and stored in $I^p$, a Maximum Intensity Projection is applied on the $I^p$ stack. In our implementation of VolumePeeler, FIJI’s native functions were used.

As it is very common used in biological applications, we adapted our method to handle time series of z-stacks. For the spherical projection we fit independent spheres (center position, radius) at each time point. For the spline projection, the user must use a minimum of two frames for control points, and by interpolation, cover the full time series. Also, for the spline projection, multi-channel stacks are supported (see user options in Additional file 1: Note 2 and Additional file 1: Fig. 3).

## Results

To assess the effectiveness of our approach we applied VolumePeeler to images of a synthetic volume and three biological models.

### Synthetic volume

**Fig. 2:**
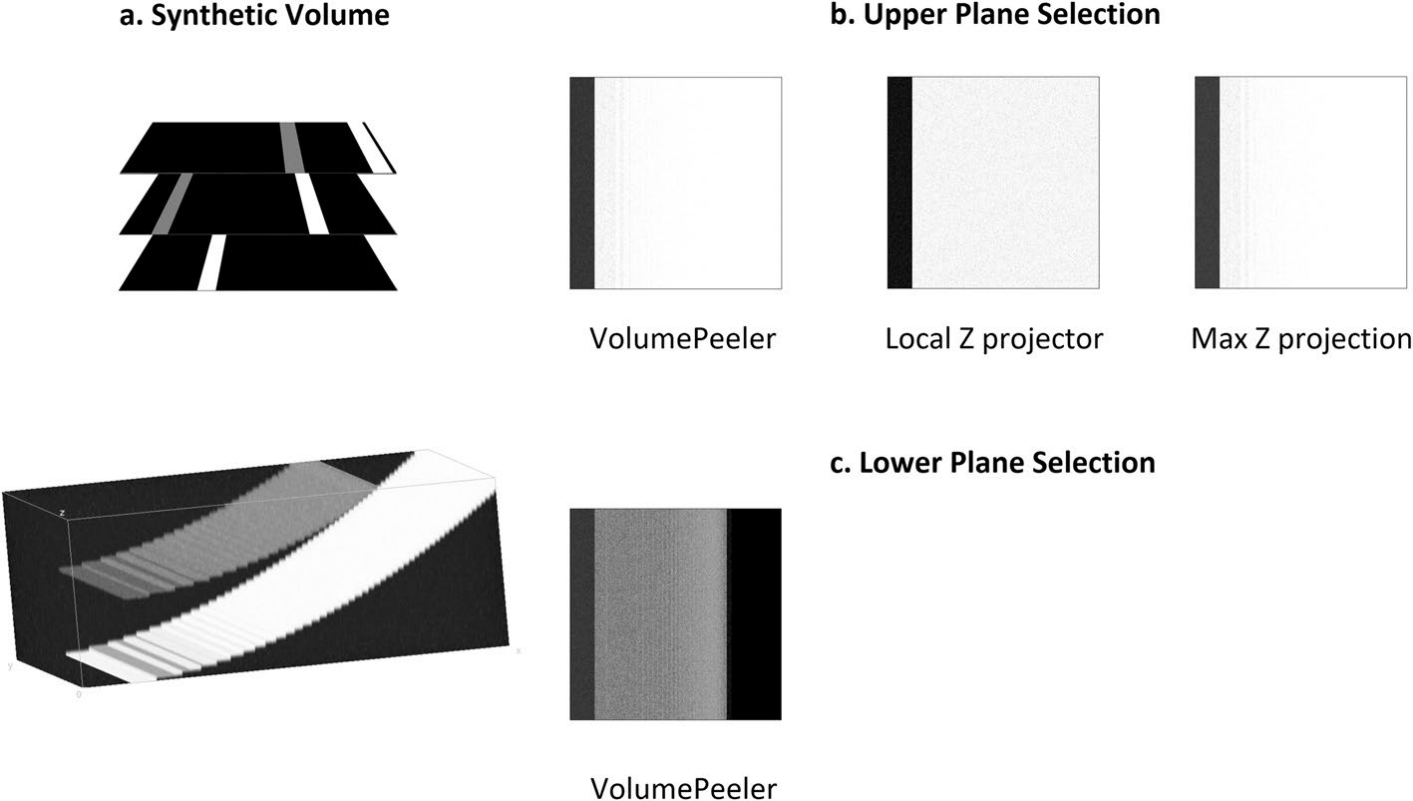
**a** Synthetic test volume. **b** VolumePeeler (proposed, $RMSE=20.54$), LocalZProjector ($RMSE=18.09$, $time=20$ [s]), and Max. Intensity ($RMSE=23.1$, $time < 1$ [s]) projections applied to the synthetic volume. VolumePeeler was set to retrieve upper plane. **c** VolumePeeler is the only method that can be set to retrieve lower gray plane ($RMSE=197.87$)

A simple volume of size $w\times h \times d = 512\times 512\times 50$ voxels was defined with two surfaces: $S_1$ and $S_2$ (Figure 2a). $S_1$ is defined as $Z_i=a X_{i}^2$. $S_2$ is defined as $Z_i=a X_{i}^2 + b$, with $a=d/h^2$ and $b=d/2$ for convenience. Voxels near $S_1$ were set to 255, and voxels near $S_2$, to 128. Additive Gaussian noise was added with mean and variance set to 0.01.

To quantify reconstruction error, we computed the Root Mean Squared Error (RMSE) in the projected 2D images. In the case of $S_1$ we define,

2 $$\begin{aligned} RMSE=\sqrt{ \sum _i (255-I(X_i,Y_i))^2 / N}, \end{aligned}$$

and, for $$S_2$$,

3 $$\begin{aligned} RMSE=\sqrt{ \sum _i (128-I(X_i,Y_i))^2 / N}. \end{aligned}$$

In both cases of $S_1$ and $S_2$, *N* is the number of pixels (projected voxels) where the retrieved plane is defined.

As shown in Figure 2b, when $S_1$ with the brighter voxels is selected as the surface to project, the three projections are similar. LocalZProjector achieves a better $RMSE=18.09$ ( $time=20$ [s]) than VolumePeeler with $RMSE=20.54$ ($time < 1$ [s]), and Max. Intensity projection with $RMSE=23.1$ ($time < 1$ [s]), but with a longer computation time. However, when $S_2$ with lower gray intensities is selected, only VolumePeeler can retrieve a solution ($RMSE=187.87$, see Figure 2c), and the other two approaches are hindered by the brighter $S_1$ voxels, and a comparable output cannot be achieved.

### Biological models

**Fig. 3:**
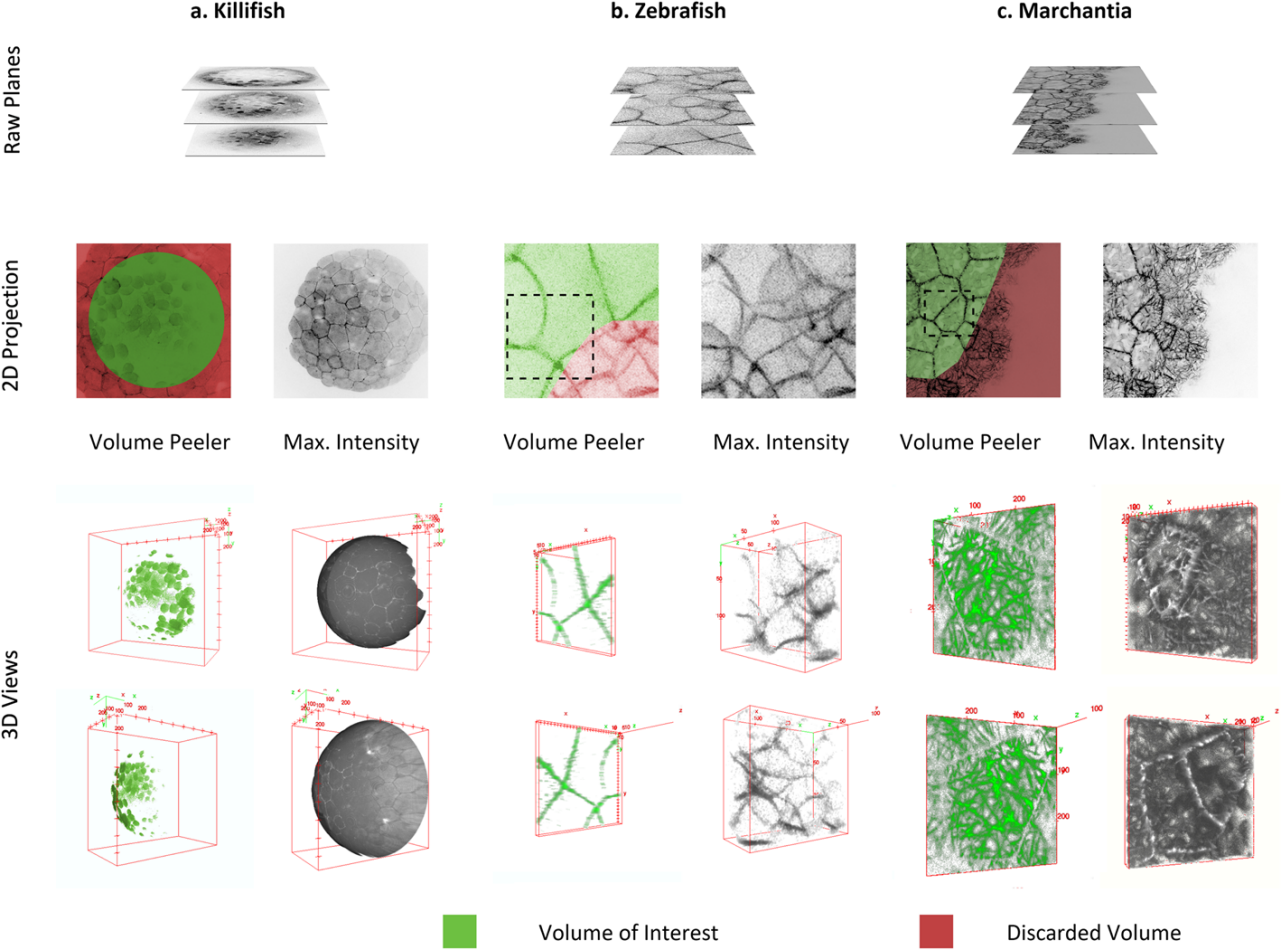
Three application examples of VolumePeeler: **a** Killifish, **b** Zebrafish, and **c** *Marchantia* tissue. In each example the stack (first row), 2D projections from VolumePeeler and Max. Intensity (second row), and 3D views (third row) are shown. Further comparisons with LocalZProjector are provided in Additional file 1: Fig. 2

The three models used are killifish, zebrafish, and *Marchantia* (Figure 3). Image acquisition details are provided in Additional file 1: Note 1. After peeling, images were segmented using a random forest approach in FIJI (Weka plugin, [8]), with Sobel and Laplacian features for the spherical annual killifish, and Gabor for the zebrafish. To measure the segmentation improvements, we applied the same segmentation to the VolumePeeler and Max. Intensity projections computing the mean Dice similarity coefficient $2|A \cap B|/(|A| + |B|)$, where *A* is the resulting segmentation and *B*, the manual (ground truth) segmentation. Dice similarity values range from 0.0 (no overlap between segmentations) to 1.0 (perfect overlap).

The killifish embryo (Figure 3a) presents two cell layers at early developmental stages: an outer epithelial layer organized as an array of packed polygonal cells, and a deeper layer of rounded mesenchymal cells that actively migrate. A conventional Max. Intensity projection applied to the image stack shows that the deeper layer of mesenchymal cells was completely hidden under the upper epithelial cell sheet (Figure 3a, second and third rows). Instead, VolumePeeler was able to remove the epithelial cell layer improving the visualization of deeper cells and making them suitable for applying additional image processing tools. After volume peeling, the Dice coefficient increased for automatic segmentation from 0.279 (derived from Max. Intensity projection) to 0.499.

Dorsal view derived from zebrafish embryo at 50% of epiboly showed two populations of cells in Figure 3b. Most external epithelia, a homologous structure described previously for killifish, and an internal group of cells called dorsal forerunner cells. A conventional Max. Intensity projection in zebrafish resulted in poorly defined and mixed boundaries between the two cell types that cannot be resolved (Figure 3b, second and third rows). However, elongated epithelial boundaries are visible once the volume is peeled. After peeling, the Dice coefficient increased for automatic segmentation from 0.4203 (derived from Max. Intensity projection) to 0.704.

*Marchantia* gemmae are reproductory structures with a lens shape and concave zones (apical notches) where dividing cells are located. The microtubule network lies beneath the surface of cells, attached to the cell membranes (Figure 3c). A simple Max. Intensity projection upon this curved morphology adds noise from the autofluorescence of chloroplasts located inside the cells, which interferes with the microtubule fluorescence (Figure 3c, second and third rows). VolumePeeler was able to remove the chloroplast signal from the deeper cell layer, and recover the microtubule signal from the upper layers.

## Conclusion

In this work we present a powerful tool aimed to ease and improve the understanding of cellular and tisular layouts that characterize embryonic development and other phenomena in 3D live imaging with optical microscopy.

In VolumePeeler we have implemented a robust and versatile algorithm to accurately render 3D image volumes into planar images. Our approach resulted in better visualization and segmentation, suitable for whole embryos and multiple tissue-derived surfaces. VolumePeeler can be applied to other tissue and cell shapes. It is available as a FIJI plugin, including support for time series and multi-channel stacks. A video tutorial is also available.

## Supplementary Information

**Additional file 1.** The file contains 2 notes, additional information on image acquisition and software interface, 3 figures with a block diagram, a visual comparison with other software, and a detailed user interface.

## Data Availability

FIJI plugin, examples, and video tutorials are openly available online from https://cimt.uchile.cl/mcerda/. Implementation is platform independent (Java), and it requires FIJI 2.3.0 or higher. Any use is allowed under MIT license. Source code is available from https://github.com/busmangit/volume-peeler. Examples and video tutorials are available under Creative Commons license (CC BY-NC).
