# Supplementary material for: VolumePeeler: a novel FIJI plugin for geometric tissue peeling to improve visualization and quantification of 3D image stacks
Source: BMC Bioinformatics. 2023 Jul 12;24:283. doi: 10.1186/s12859-023-05403-z (PMC10337153; doi:10.1186/s12859-023-05403-z)
Supplement: Supplementary file 1 — Additional file 1. The file contains 2 notes, additional information on image acquisition and software interface, 3 figures with a block diagram, a visual comparison with other software, and a detailed user interface. [file 12859_2023_5403_MOESM1_ESM.pdf]

Supplementary Material for “VolumePeeler: A novel FIJI plugin for geometric tissue peeling to improve visualization and quantification of 3D image stacks”

Marilyn Gatica[1], Carlos F. Navarro[2,3], Alejandro Lavado[2,3], Germán Reig[4], Eduardo Pulgar[5], Paula Llanos[6], Steffen Härtel[2,3,7], Andrea Ravasio[8], Cristina Bertocchi[9], Miguel L. Concha[2,3,10], and Mauricio Cerda[2,3,7,10,*]

[1]*NIHR Nottingham Biomedical Research Centre, School of Medicine, University of Nottingham, Nottingham, United Kingdom.*

[2]*Integrative Biology Program, Institute of Biomedical Sciences, Facultad de Medicina, Universidad de Chile.*

[3]*Biomedical Neuroscience Institute, Santiago, Chile.*

[4]*Escuela de Tecnología Médica and Centro Integrativo de Biología y Química Aplicada, Universidad Bernardo O'Higgins, Santiago, Chile.*

[5]*CEDAI Aquaculture, Santiago, Chile.*

[6]*Facultad de Ingeniería y Ciencias, Universidad Adolfo Ibañez, Vinña del Mar, Chile.*

[7]*Center for Medical Informatics and Telemedicine, Facultad de Medicina, Universidad de Chile, Santiago, Chile.*

[8]*Institute for Biological and Medical Engineering, Schools of Engineering, Medicine and Biological Sciences, Pontificia Universidad Católica de Chile, Santiago, Chile.*

[9]*Laboratory for Molecular Mechanics of Cell Adhesion, Department of Physiology, Faculty of Biological Sciences, Pontificia Universidad Católica de Chile, Santiago, Chile.*

[10]*Center for Geroscience, Brain Health and Metabolism, Santiago, Chile.*

[*]*Corresponding author: Mauricio Cerda, mauricio.cerda@uchile.cl*

Contents

1 Supplementary Note 1. Image acquisition. 2

2 Supplementary Note 2. Spline projection User Interface. 2

3 Supplementary Figures. 3

1 Supplementary Note 1. Image acquisition.

Annual killifish embryos (*Austrolebias nigripinnis*) expressing GAP43-EGFP were mounted in a custom-designed chamber filled with dissolved 1% low-melting-point agarose in ERM rearing medium and placed on the microscope stage, between 48 and 72 hours post-fertilization. Zebrafish (*Danio rerio)* wild-type AB embryos injected with 50 pg of gap43-RFP mRNA were mounted in 0.5% low melting point agarose in a custom-designed chamber at 50% epiboly stage. *In vivo* confocal microscopy was performed in a Leica TCS LSI microscope (Leica Microsystems, Germany) using a 5x objective for killifish and zebrafish embryo. *Marchantia* of expressing GFP-MpTUB1 and Lit6b-mCitrin transgenic lines were mounted in a custom chamber with Gambor B5 agar media. Microscopy was performed using a Leica SP8 upright microscope (Leica Microsystems, Germany) equipped with a 40x objective.

2 Supplementary Note 2. Spline projection User Interface.

The interface for spline projection has several features that allow for an efficient and user-friendly experience, as shown in Supplementary Figure 3. To summarize, the interface allows: 3x3, 4x4, or 5x5 control points and channel selection, save/load z-values, repeat z-values, interpolate z-values, selection of peeling behavior (upper, lower, band), and visualization options (preview, process, adjust brightness).

3 Supplementary Figures.

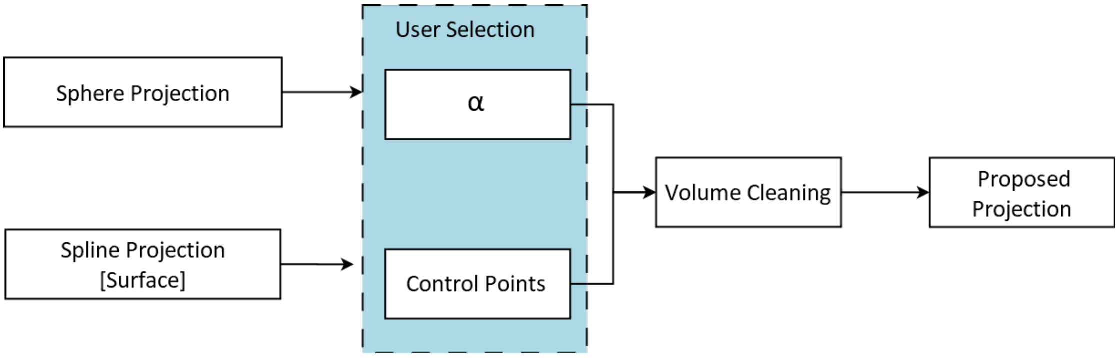

Supplementary Figure 1: Block diagram of the VolumePeeler approach.

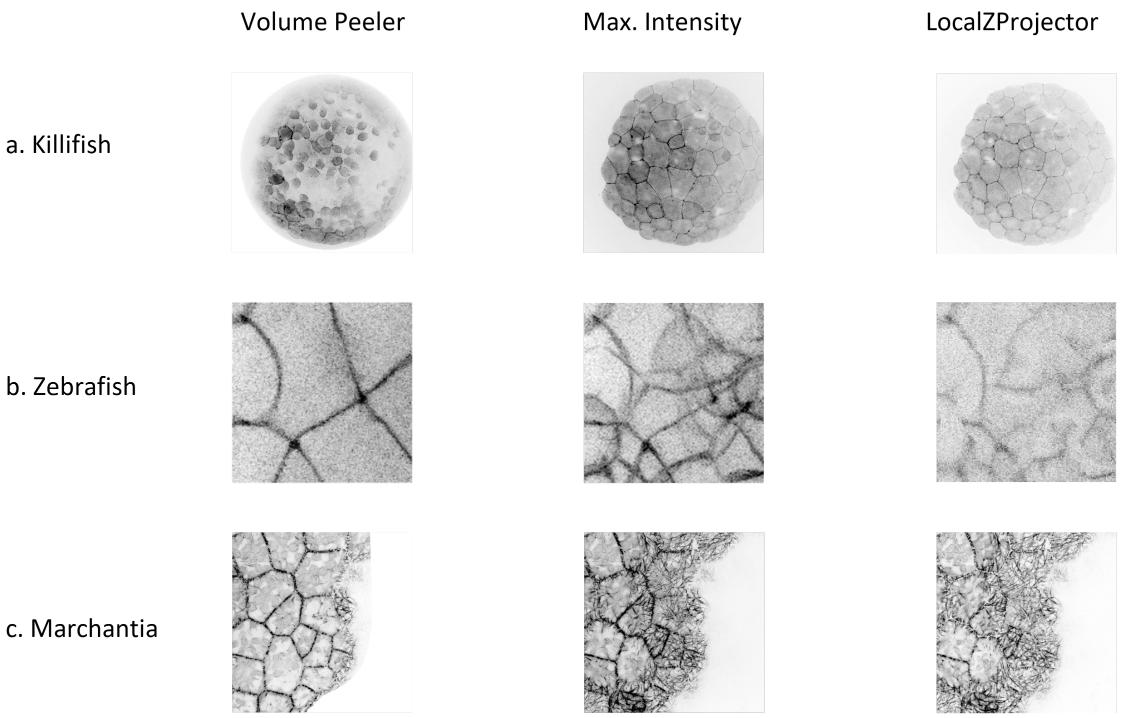

Supplementary Figure 2: Visual comparison of VolumePeeler (proposed approach), Maximum Intensity Projection, and LocalZProjector, for three biological model examples: **a** killifish, **b** zebrafish, **c** *Marchantia*.

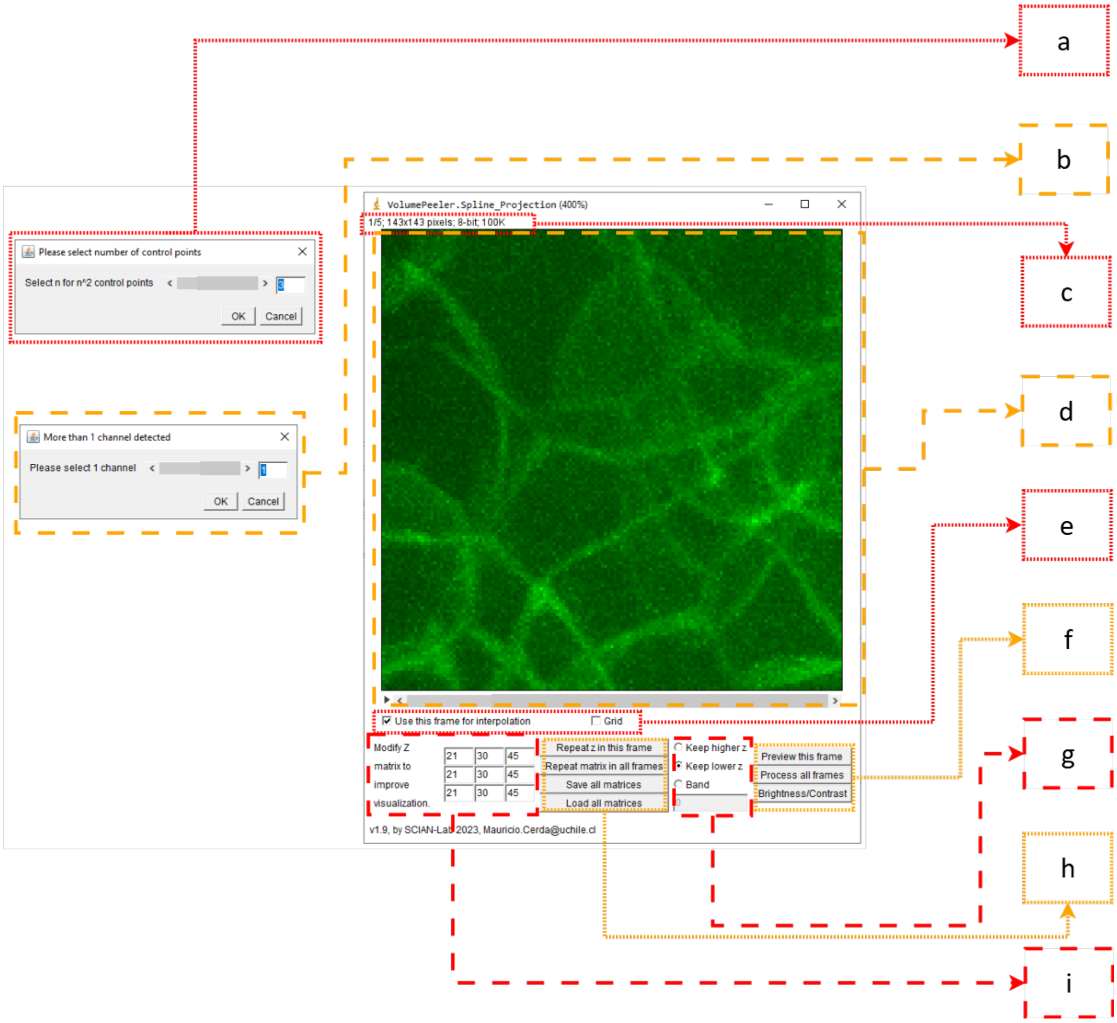

Supplementary Figure 3: Spline projection user interface. **a** Selector for number of control points. **b** Selector of working channel. **c** Stack information label. **d** Preview window. **e** Frame selection option for interpolation and grid lines (on/off) control. **f** Preview, process, and brightness/contrast control buttons. **g** Volume peeling behavior selector. **h** Shortcut buttons for cloning and saving/loading values and matrices. **i** Depth matrix control points to define the reference surface.
